# A high-density genetic map constructed using specific length amplified fragment (SLAF) sequencing and QTL mapping of seed-related traits in sesame (*Sesamum indicum* L.)

**DOI:** 10.1186/s12870-019-2172-5

**Published:** 2019-12-27

**Authors:** Hua Du, Haiyang Zhang, Libin Wei, Chun Li, Yinghui Duan, Huili Wang

**Affiliations:** Henan Sesame Research Center, Henan Academy of Agricultural Sciences, Zhengzhou, Henan 450002 People’s Republic of China

**Keywords:** QTL mapping, Seed traits, Seed size, Seed coat color, Sesame, SLAF, Single nucleotide polymorphism

## Abstract

**Background:**

Sesame (*Sesamum indicum* L., 2*n* = 2*x* = 26) is an important oilseed crop with high oil content but small seed size. To reveal the genetic loci of the quantitative seed-related traits, we constructed a high-density single nucleotide polymorphism (SNP) linkage map of an F_2_ population by using specific length amplified fragment (SLAF) technique and determined the quantitative trait loci (QTLs) of seed-related traits for sesame based on the phenotypes of F_3_ progeny.

**Results:**

The genetic map comprised 2159 SNP markers distributed on 13 linkage groups (LGs) and was 2128.51 cM in length, with an average distance of 0.99 cM between adjacent markers. QTL mapping revealed 19 major-effect QTLs with the phenotypic effect (R^2^) more than 10%, i.e., eight QTLs for seed coat color, nine QTLs for seed size, and two QTLs for 1000-seed weight (TSW), using composite interval mapping method. Particularly, LG04 and LG11 contained collocated QTL regions for the seed coat color and seed size traits, respectively, based on their close or identical locations. In total, 155 candidate genes for seed coat color, 22 for seed size traits, and 54 for TSW were screened and analyzed.

**Conclusions:**

This report presents the first QTL mapping of seed-related traits in sesame using an F_2_ population. The results reveal the location of specific markers associated with seed-related traits in sesame and provide the basis for further seed quality traits research.

## Background

Sesame (*Sesamum indicum* L.), a diploid species (2*n* = 2*x* = 26), is one of the most ancient and important domestic oilseed crops, with small genome size of 354 Mb [1, 2]. Sesame seed is a nutritious source owing to high content of unsaturated fatty acids and natural antioxidants (such as sesamin and sesamol) [3, 4]. Sesame has now been widely cultivated in many tropical and subtropical countries of Asia, Africa, and Southern America, with an average annual production of 5.82 million tons from 2011 to 2016 (FAO data) produced globally over a harvest area of 9.81 million hectares. Sesame has high tolerance to drought and readily adapts to various soil types and cultivation practices. However, its production, due to the small size of its seeds, faces challenges similar to many crops such as lowered germination rate, slow growth, and low tolerance of the seedlings to environmental conditions, which eventually affects crop yield [5–8]. Therefore, both increasing the seed size and improving the seed quality are important aspects of sesame production.

In crops, seed size is a complex quantitative trait often described using three indicators: seed length, seed width, and seed thickness [9–11]. Till now, many quantitative trait loci (QTLs) or genes related to seed size traits have been reported in rice [12], maize [13], and wheat [5]. In tomato, *fw2.2* gene is an important locus affecting fruit weight and acting as a negative regulator of cell division in carpels [14, 15]. Other quantitative seed-related traits include seed weight and seed coat color. In sesame, the value of 1000-seed weight (TSW) varies from 1.82 g to 4.74 g [16]. In recent years, five candidate genes associated significantly with oilseed yield were detected using genome wide association study (GWAS) [17]. The most common colors for mature sesame seeds are black and pure white, and the less common shades are gray, brown, golden, yellow, light white and so on. The seed coat color trait for sesame varieties is related to seed biochemical characteristics, antioxidant capacity, and even disease resistance, and is considered to be a sign of species evolution within genus *Sesamum* [18–22]. Until now, four QTLs for seed coat color were detected using an F_2_ population [23]. A total of 32 candidate genes near seed coat color loci were determined according to GWAS [24]. However, to the best of our knowledge, there are no reports on the location of QTL and genes related to seed size traits in sesame, and genes controlling seed coat color and seed weight are yet to be identified in this species. Discovery of QTL and genes related to seed size, seed coat color, and seed weight is requisite for developing sesame molecular breeding techniques.

The development of next-generation sequencing (NGS) makes it possible to rapidly construct high-density or ultra-dense single nucleotide polymorphism (SNP) genetic maps for map-based gene identification in a number of crops [25–28]. For sesame, six high-density molecular genetic maps have been constructed and are used for sesame genome assembly and map-based gene cloning [23, 24, 29–33]. Especially, the construction of an ultra-dense SNP genetic map using the whole genome re-sequencing approach enhances gene cloning and genomics research in sesame [32, 34]. Based on the linkage mapping method and candidate variants screening, two sesame genes, *Sidt1* controlling inflorescence determinacy and *Sicl1* controlling leaf curling and capsule indehiscence, were successfully cloned [32, 34].

To clarify the genetic mechanisms of seed-related traits in sesame, we genetically characterized seed size, TSW, and seed coat color traits in an F_3_ population. The dominant QTLs related to seed size, seed weight, and seed coat color traits were determined in sesame based on the high-density genetic map constructed using specific length amplified fragment sequencing (SLAF-seq) approach, and the candidate sesame genes in QTL intervals were analyzed. The results provide a solid foundation for further genetic regulation analysis of seed-related traits in sesame.

## Results

### Phenotypic analysis of seed-related traits in F_3_ progeny under two environments

**Fig. 1:**
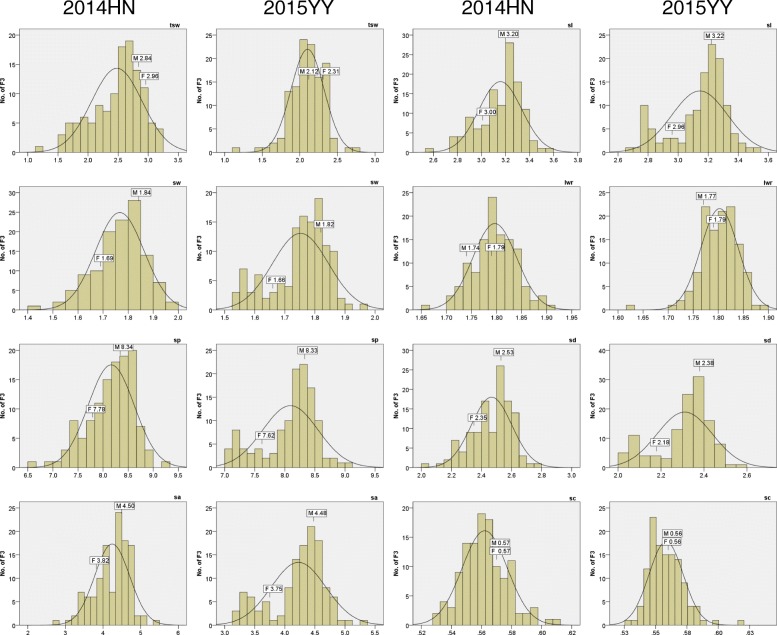
Distributions of the phenotypic data in the F_3_ population from the cross of ‘Gaoyou 8’ and ‘Ganzhi 6’. tsw, thousand seed weight; sl, seed length; sw, seed width; lwr, length-to-width ratio; sp., seed perimeter; sd, seed diameter; sa, seed area; sc, seed circularity. HN, Hainan field trial location; YY, Yuanyang field trial location. Mean of two parents is indicated at the top of each histogram, with *M* and *F* representing Ganzhi 6 (male parent) and Gaoyou 8 (female parent), respectively

**Fig. 2:**
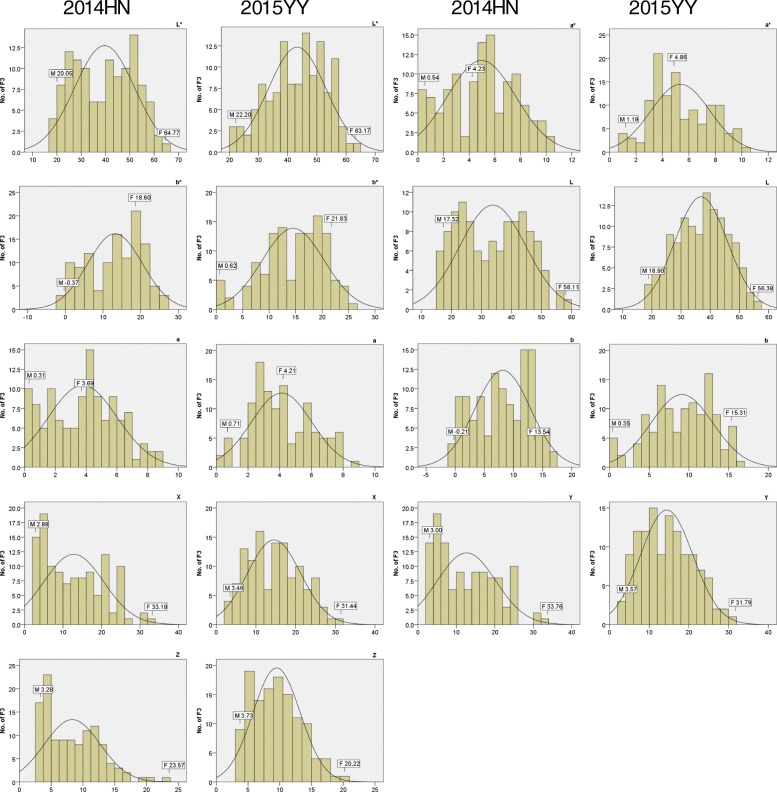
Distributions of the phenotypic data in the F_3_ population from the cross of ‘Gaoyou 8’ and ‘Ganzhi 6’. L*, a*, b*, L, a, b, X, Y, Z, different traits of sesame seed coat color. HN, Hainan field trial location; YY, Yuanyang field trial location. Mean of two parents is indicated at the top of each histogram, with *M* and *F* representing Ganzhi 6 (male parent) and Gaoyou 8 (female parent), respectively

In sesame, the appearance quality of seed is involved in seed size, seed weight, and seed coat color. To reveal the genetic basis of the above seed-related traits, we constructed a cross population derived from two sesame cultivars, Gaoyou 8 (female parent, white seeded) and Ganzhi 6 (male parent, black seeded). The phenotypic variation of 17 indicators related to seed size, TSW, and seed coat color traits in the F_3_ generation and two parents grown in two different environments was investigated (Figs. 1 and 2; Table 1).

**Table 1:** Statistics analysis of seed-related traits of the parents and F_3_ population

Trait		Environment	Parent		F_3_				
			Ganzhi 6 (M)	Gaoyou 8 (F)	Mean	Range	*CV* (%)	Skewness	Kurtosis
Seed coat color	L*	Hainan	20.05	64.77	39.57 ± 12.03	18.49–62.19	30.40	−0.05	−1.24
		Yuanyang	22.20	63.17	43.25 ± 9.54	21.63–60.86	22.05	−0.28	−0.74
	a*	Hainan	0.54	4.23	5.00 ± 2.65	0.08–10.46	53.01	−0.05	−0.87
		Yuanyang	1.19	4.86	5.39 ± 2.22	0.81–10.58	41.19	0.25	−0.71
	b*	Hainan	−0.37	18.60	13.26 ± 7.18	−0.20-27.24	54.10	−0.30	−0.95
		Yuanyang	0.62	21.83	14.59 ± 5.70	0.48–25.36	39.07	−0.42	−0.39
	L	Hainan	17.32	58.11	33.74 ± 10.71	16.21–55.77	31.74	0.07	−1.22
		Yuanyang	18.90	56.38	36.86 ± 8.70	18.47–53.93	23.60	−0.14	−0.84
	a	Hainan	0.31	3.69	3.81 ± 2.24	0.04–8.81	58.83	0.13	−0.83
		Yuanyang	0.71	4.21	4.14 ± 1.90	0.49–8.77	45.89	0.33	−0.69
	b	Hainan	−0.21	13.54	8.18 ± 4.69	−0.11–17.15	57.29	−0.16	−1.07
		Yuanyang	0.35	15.31	9.14 ± 3.81	0.27–16.64	41.68	−0.30	− 0.59
	X	Hainan	2.88	33.19	12.70 ± 7.55	2.54–26.07	59.47	0.41	−0.98
		Yuanyang	3.46	31.44	14.50 ± 6.47	3.29–28.83	44.62	0.24	−0.88
	Y	Hainan	3.00	33.76	12.53 ± 7.38	2.63–31.11	58.59	0.44	−0.91
		Yuanyang	3.57	31.79	14.35 ± 6.36	3.41–29.09	44.32	0.26	−0.83
	Z	Hainan	3.28	23.57	8.23 ± 4.16	2.78–20.40	50.53	0.63	−0.40
		Yuanyang	3.73	20.22	9.47 ± 3.58	3.58–18.33	37.80	0.37	−0.64
Seed size	length (sl)	Hainan	3.20	3.00	3.16 ± 0.18	2.59–3.54	5.64	−0.70	0.15
		Yuanyang	3.22	2.96	3.15 ± 0.19	2.69–3.50	5.91	−0.84	−0.03
	width (sw)	Hainan	1.84	1.69	1.76 ± 0.10	1.45–2.00	5.49	−0.60	0.50
		Yuanyang	1.82	1.66	1.75 ± 0.09	1.53–1.97	5.31	−0.68	−0.13
	length-to-width ratio (lwr)	Hainan	1.74	1.79	1.80 ± 0.04	1.66–1.90	2.45	−0.03	0.29
		Yuanyang	1.77	1.79	1.80 ± 0.04	1.63–1.89	2.11	−0.95	3.23
	perimeter (sp)	Hainan	8.34	7.78	8.16 ± 0.46	6.62–9.19	5.63	−0.71	0.39
		Yuanyang	8.33	7.62	8.10 ± 0.46	7.01–9.02	5.68	−0.85	−0.01
	diameter (sd)	Hainan	2.53	2.35	2.47 ± 0.13	2.04–2.77	5.43	−0.66	0.20
		Yuanyang	2.38	2.18	2.31 ± 0.13	2.00–2.59	5.54	−0.82	−0.03
	area (sa)	Hainan	4.50	3.82	4.24 ± 0.47	2.77–5.40	10.96	−0.60	0.20
		Yuanyang	4.48	3.75	4.23 ± 0.45	3.18–5.27	10.74	−0.73	−0.05
	circularity (sc)	Hainan	0.57	0.57	0.56 ± 0.02	0.53–0.61	2.67	0.42	0.34
		Yuanyang	0.56	0.56	0.56 ± 0.01	0.53–0.62	2.51	0.99	2.39
Thousand seed weight		Hainan	2.84	2.96	2.47 ± 0.42	1.18–3.19	16.94	−0.67	−0.07
		Yuanyang	2.12	2.31	2.10 ± 0.22	1.17–2.77	10.57	−0.50	2.57

*Seventeen traits are listed. M, male parent; F, female parent

Analysis of the seven indicators related to seed size showed that the values of SA, SL, SP, SD, and SW obviously fluctuated based on the coefficient of variation (*CV*) across the two field trials among the 122 progeny lines. A positive transgressive segregation was observed for LWR in F_3_ population. Furthermore, variance analysis indicated that the absolute values of skewness and kurtosis of SL, SW, SP, SD, and SA traits were < 1 at the two environments, and those for LWR and SC were < 1 in HN (2014) (Table 1). The results were mostly consistent with the frequency distribution results, except for SP, SD, and SA. The near-normal curve distribution of SL, SW, LWR, and SC using the variance analysis and frequency distribution suggested a polygene mode of the genetic control, whereas SP, SA, and SD showed a bimodal distribution in the frequency distributions analysis, suggesting the involvement of major effect genes (Fig. 1; Table 1).

To reveal the relationship of the above seed-related traits, we conducted the correlation coefficient analysis for the F_3_ population in the two field trials (Additional file 1: Table S1). As to seed size, significant positive correlations (*P* ≤ 0.01) between SL, SW, SP, and SA were observed. The values of SD significantly and positively correlated with SL, SP, and SA, whereas SC significantly and negatively correlated with SL, SP, SA, and SD (P ≤ 0.01). However, the correlation of some indicators was affected by environment. For example, LWR significantly positively correlated (P ≤ 0.01) with SP, SD, and SA, whereas SW was significantly negatively correlated with SC at the YY location (P ≤ 0.01).

Meanwhile, the absolute values of skewness and kurtosis of TSW were < 1 at the HN station (2014) (Table 1). The near-normal frequency curve distribution indicated that the trait was controlled by polygenes (Fig. 1). The values of TSW also varied significantly with negative transgressive segregation presented in the F_3_ population in the two field trials (HN, 1.18–3.19 g; YY, 1.17–2.77 g) ,although the values of the two parents were similar (Table 1). In addition, the correlational relationship between TSW and the seven seed size traits was also influenced by the environment. Except for LWR, all the seed size traits significantly positively correlated (*P* ≤ 0.01) with TSW in the HN location, while SL, SW, SP, SD, and SA negatively correlated (*P* ≤ 0.05) with TSW in YY. The significant negative correlation (P ≤ 0.01) between LWR and TSW existed only in the HN treatment (Additional file 1: Table S1).

For seed coat color trait, the values of L*, a*, and b* in the two parents Gaoyou 8 and Ganzhi 6 and the F_3_ individuals varied significantly (Table 1). The L* values in the F_3_ population ranged from 18.49 to 62.19; a* and b* values ranged from 0.08 to 10.46 and from − 0.20 to 27.24, respectively, at the HN site. Trial-wide correlation coefficients of seed coat color spaces were all significantly positive at the level of P ≤ 0.01 (Additional file 2: Table S2). In the F_3_ population, the seed coat color spaces were exhibited in a typical quantitative manner and showed a bimodal distribution (Fig. 2). The data suggested that black, white, and yellow were predominant in the sesame seed coat color spaces in the F_3_ population.

### Analysis of SLAF-seq data and SLAF marker identification

To locate the gene or marker sites for the seed-related traits in a high-density SNP genetic map, we sequenced the 122 F_2_ individuals and the parents Gaoyou 8 (white seeded) and Ganzhi 6 (black seeded) using the high-throughput sequencing method. As a result, a total of 26.31 Gb genome data was obtained, and high sequencing data with the Q30 ratio comprised 82.98% (Additional file 3: Table S3). A 35.97-fold and 37.60-fold coverage was obtained for Ganzhi 6 and Gaoyou 8, respectively. The genome coverage of Illumina data for the F_2_ individuals ranged from 1.97-fold to 9.29-fold, with an average of 5.17-fold.

Based on the sesame reference genome sequences (http://ocri-genomics.org/Sinbase_v2.0), 123,679 and 133,354 SLAF markers were found specific for Ganzhi 6 (male parent) and Gaoyou 8 (female parent), respectively. For the 122 F_2_ individuals, the number of SLAF markers of each sample ranged from 50,181 to 113,301, with an average of 95,887 (Additional file 3: Table S3). Of the detected 141,313 high-quality SLAFs, 9134 were polymorphic between the two parents, with a polymorphism ratio of 6.46% (Table 2). Of these, 8159 polymorphic markers were classified into eight segregation patterns.

**Table 2:** Identification of SLAF markers of the F_3_ population derived from the cross of ‘Gaoyou 8’ and ‘Ganzhi 6’

Item	Polymorphic type	Non-polymorphic type	Repetitive type	Total number
Number of SLAF markers	9134	132,028	151	141,313
Number of reads	4,660,351	66,728,794	29,937	71,419,082
Percentage (%)	6.46	93.43	0.11	100.00

Before constructing the genetic map, the homozygous markers with different genotypes in the two parents were screened. According to the aa × bb segregation pattern in the F_2_ population, 6682 markers were finally chosen for SNP genetic map construction. Of the 6682 markers, 2650 presented the sequence depth of > 10-fold and > 2-fold in the two parents and the progeny, respectively. All these 2650 markers comprised more than 70% integrity of SLAF tags.

### High-density genetic map construction and structure analysis

**Fig. 3:**
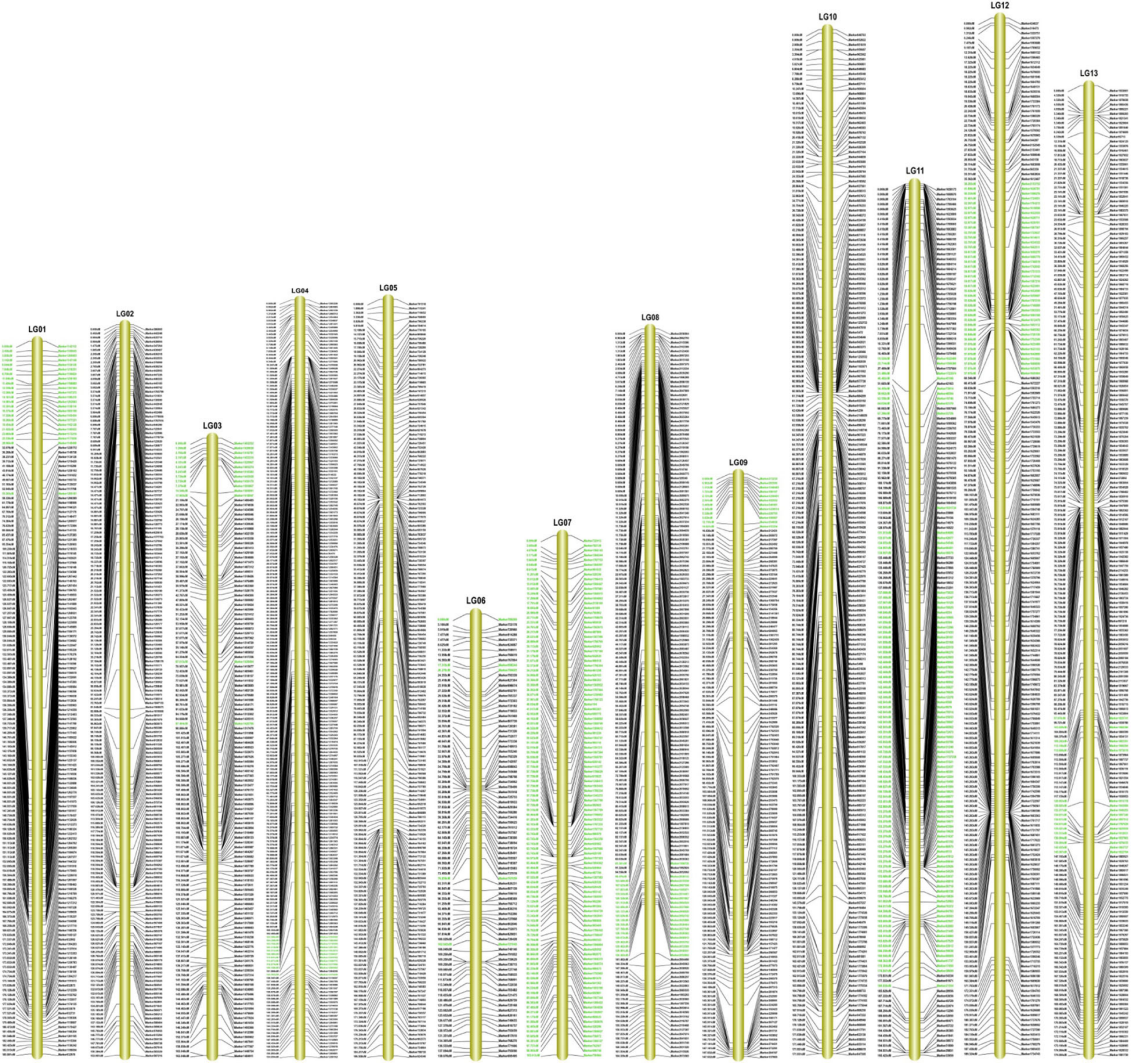
13 linkage groups of high-density linkage map for sesame. Segregation distortion markers on the map are highlighted in green

Finally, 2159 of the 2650 markers were mapped onto the 13 LGs (Fig. 3; Table 3). All the markers on the map presented a high average integrity of 93.15%. For the SLAF molecular genetic map, the total length of the 13 LGs was 2128.51 cM (Table 3). The largest LG (195.42 cM) was LG11, which anchored 175 markers, while the smallest LG (103.01 cM) was LG07 with 108 markers. The average LG length was 166.00 cM, and the average marker density was 0.99 cM/SLAF marker. The degree of linkage between markers reflected by ‘Gap ≤ 5’ ranged from 95.40 to 100.00%, with the mean of 97.86%. The largest gap on this map was located in LG11, with the length of 15.00 cM.

**Table 3:** Linkage group information of the genetic map in sesame

Linkage group ID	Number of markers				Total distance (cM)	Average distance (cM)	Max gap (cM)	Gaps < =5
	Total	SNP_only	InDel_only	SNP&InDel				
LG01	155	155	0	0	185.80	1.21	13.68	98.70%
LG02	176	176	0	0	154.00	0.88	8.29	96.00%
LG03	130	129	1	0	163.05	1.26	7.16	97.67%
LG04	221	218	2	1	154.09	0.70	7.61	99.09%
LG05	174	172	0	2	186.94	1.08	5.02	99.42%
LG06	87	87	0	0	138.68	1.61	6.46	97.67%
LG07	108	108	0	0	103.01	0.96	4.11	100.00%
LG08	166	163	2	1	164.56	1.00	11.10	96.97%
LG09	134	133	1	0	147.63	1.11	9.63	96.99%
LG10	210	209	1	0	171.93	0.82	6.51	98.56%
LG11	175	174	0	1	195.42	1.12	15.00	95.40%
LG12	213	213	0	0	189.32	0.89	8.44	97.64%
LG13	210	205	1	4	174.08	0.83	7.97	98.09%
Maximum	221	218	2	4	195.42	1.61	15.00	100.00%
Minimum	87	87	0	0	103.01	0.70	4.11	95.40%
Total	2159	2142	8	9	2128.51	/	/	/
Average	166.08	164.77	0.62	0.69	166.00	/	/	97.86%

For the SLAF genetic map, there were three types of markers, i.e., 2142 (99.21%) ‘SNP_only’ markers, 8 (0.37%) ‘InDel_only’ (insertion-deletion) markers, and 9 (0.42%) ‘SNP&InDel’ markers (Table 3). Of the predominant 2142 ‘SNP_only’ markers, 739 (34.5%) had more than two SNP loci, while the other 1403 (65.5%) markers presented single SNP locus. In total, 3129 SNP loci were detected among the 2159 markers (Tables 3 and 4). The transition types of R (A/G) (32.85%) and Y (C/T) (30.43%) were the main SNP alleles, followed by four transversion types, i.e., M (A/C), K (G/T), S (C/G), and W (A/T). The percentages of the above four transversion types ranged from 8.44 to 10.13% and accounted for 36.72% of all SNPs.

**Table 4:** Statistic analysis of mapped SNP marker types in SLAF map

Type	Number	Ratio
W(A/T)	317	10.13%
R(A/G)	1028	32.85%
M(A/C)	264	8.44%
K(G/T)	276	8.82%
Y(C/T)	952	30.43%
S(C/G)	292	9.33%
Total	3129	100.00%

### Segregation distortion of SNP markers on the high-density genetic map

Statistics results showed that a total of 343 markers on the genetic map exhibited the significance of segregation distortion (*P* < 0.05) (Table 5). All these SNP markers belonged to ‘SNP_only’ type and distributed mostly on the ends of LGs, except for the smallest LG (LG07). The segregation distortion frequency of markers on the largest LG (LG11) and the smallest LG (LG07) was 53.14 and 100.00%, respectively. In addition, 11 SDRs were detected on nine LGs, and LG11 had the largest number of SDRs (Table 5).

**Table 5:** Distribution of segregation distortion markers

Linkage Group ID	All Marker		Segregation distortion marker		*X*^2^	*P*	Frequency of segregation distortion marker	SDR number
	Number	Percentage	Number	Percentage				
LG01	155	7.18	23	6.71	5.93	0.11	14.84	1
LG02	176	8.15	0	0.00	1.62	0.50	0.00	0
LG03	130	6.02	14	4.08	9.12	0.20	10.77	1
LG04	221	10.24	11	3.21	1.97	0.54	4.98	1
LG05	174	8.06	0	0.00	2.01	0.48	0.00	0
LG06	87	4.03	5	1.46	3.20	0.38	5.75	0
LG07	108	5.00	108	31.49	190.42	0.00	100.00	1
LG08	166	7.69	20	5.83	13.24	0.21	12.05	1
LG09	134	6.21	12	3.50	7.09	0.32	8.96	1
LG10	210	9.73	0	0.00	2.09	0.41	0.00	0
LG11	175	8.11	93	27.11	8.92	0.09	53.14	3
LG12	213	9.87	40	11.66	15.24	0.26	18.78	1
LG13	210	9.73	17	4.96	4.21	0.40	8.10	1
Total	2159		343				15.89	11

* *X*^2^ and *P* indicate *X*^2^ values with one degree of freedom and the corresponding probability, respectively

SDR refers to segregation distortion region

### QTL location of seed-related traits in two environments

Based on the above phenotypic data and the LGs, we determined the QTLs of seed size, seed weight, and seed coat color traits using the composite interval mapping methods (Table 6). A total of 31 seed-related QTLs (R^2^ ≥ 5%) were located in eight linkage groups, and a range of one to three QTLs were detected for individual traits (Table 6). Of these, 14 QTLs for seed coat color traits were located in LG04, LG09, and LG12. Fourteen QTLs related to the seed size traits were located in five LGs. For TSW, there were three QTLs found in LG04, LG09, and LG12. Of the 31 QTLs, 19 major-effect QTLs were detected with R^2^ ≥ 10%, and 10 QTLs (R^2^ ≥ 10%) were detected with similar QTL regions (Table 6).

**Table 6:** QTL location of the seed-related traits in the F_3_ population from the cross of ‘Gaoyou 8’ and ‘Ganzhi 6’

Trait	QTL	Linkage Group ID	QTL region (cM)	Left marker	Right marker	LOD	Additive	Dominance	D/A	GAM	R^2^ (%)	HN	YY
Seed coat color	qsccL*4	LG04	78.19–78.68	MK1281005	MK1338566	5.93	4.4202	−4.0482	0.9158	D	8.56	√	
	*qsccL4*	LG04	78.19–78.68	MK1281005	MK1338566	4.25	3.3711	−1.3881	0.4118	PD	7.48		√
	qsccb*4	LG04	78.19–78.68	MK1281005	MK1338566	8.58	4.4717	−3.6157	0.8086	PD	23.10	√	
	qscca*4	LG04	79.58–79.99	MK1269895	MK1382037	11.73	1.5665	−1.0255	0.6546	PD	19.09	√	
	*qscca4–2*	LG04	79.58–79.99	MK1269895	MK1382037	15.01	1.1743	−0.6035	0.5139	PD	19.79		√
	*qsccL4*	LG04	80.89–81.38	MK1303398	MK1353258	6.21	3.7529	−3.3038	0.8803	D	7.62	√	
	*qscca4–1*	LG04	80.89–81.38	MK1303398	MK1353258	11.92	1.2502	−0.8732	0.6984	PD	17.43	√	
	*qsccb4*	LG04	80.89–81.38	MK1303398	MK1353258	12.44	2.5780	−2.0005	0.7760	PD	17.65	√	
	*qsccX4*	LG04	80.89–81.38	MK1303398	MK1353258	6.17	2.8449	2.3046	0.8101	D	8.27	√	
	*qsccY4*	LG04	80.89–81.38	MK1303398	MK1353258	5.91	2.6292	−2.1740	0.8269	D	7.63	√	
	qscca*9	LG09	80.47–90.56	MK1792520	MK290567	10.50	1.3793	−0.6402	0.4641	PD	20.02		√
	*qsccY9*	LG09	101.72–103.68	MK183845	MK253698	10.79	5.2956	−1.1719	0.2213	PD	33.25		√
	*qsccZ9*	LG09	90.56–90.97	MK1791984	MK253852	4.83	3.3809	0.5082	0.1503	A	32.88	√	
	*qsccZ12*	LG12	159.98–161.95	MK1580955	MK1696180	2.72	0.9185	1.0133	1.1032	D	5.58		√
Seed size	*qsa1*	LG01	36.27–41.19	MK1149758	MK1110290	2.10	−0.1220	0.1745	1.4303	OD	6.64		√
	*qsw1*	LG01	36.27–39.71	MK1149758	MK1219420	2.07	−0.0257	0.0353	1.3735	OD	6.73		√
	*ql/w1*	LG01	172.85–173.34	MK1394203	MK1213353	6.09	−0.0169	0.0130	0.7692	PD	11.76		√
	*qsc1*	LG01	183.42–183.92	MK1130242	MK1199094	4.89	0.0068	−0.0024	0.3529	PD	12.16		√
	*ql/w2*	LG02	16.77–17.26	MK111106	MK635618	3.26	−0.0211	0.0067	0.3175	PD	11.43	√	
	*qsc2*	LG02	16.77–17.26	MK111106	MK635618	4.19	0.0075	0.0026	0.3467	PD	12.11	√	
	*qsw5*	LG05	22.27–22.27	MK714015	MK804273	2.18	0.0379	0.0145	0.3826	PD	8.97		√
	*qsd5*	LG05	22.27–22.27	MK714015	MK804273	3.34	0.0441	0.0179	0.4059	PD	6.43		√
	*qsp11*	LG11	164.51–165.41	MK84783	MK21445	5.52	−0.199	−0.1834	0.9216	D	13.97	√	
	*qsa11*	LG11	165.41–166.23	MK21445	MK39038	5.14	−0.2009	−0.184	0.9159	D	13.86	√	
	*qsl11*	LG11	165.41–166.23	MK21445	MK39038	4.66	−0.0784	−0.0771	0.9834	D	15.12	√	
	*qsw11*	LG11	165.41–166.23	MK21445	MK39038	3.60	−0.0401	−0.0349	0.8703	D	12.39	√	
	*qsd11*	LG11	165.41–166.23	MK21445	MK39038	4.78	−0.0593	−0.0552	0.9309	D	14.70	√	
	*qsl13*	LG13	66.41–66.90	MK1860058	MK1843260	2.94	−0.0673	0.0489	0.7266	PD	7.45		√
Thousand seed weight	*qtsw4*	LG04	57.19–57.69	MK1268296	MK1268983	6.70	−0.2354	0.0448	0.1903	A	15.09	√	
	*qtsw9*	LG09	120.45–120.86	MK193210	MK167922	3.72	−0.1228	0.1556	1.2671	OD	6.90	√	
	*qtsw12*	LG12	124.45–124.92	MK1695007	MK1754691	3.78	0.1417	−0.0054	0.0381	A	19.56		√

*Seed coat color traits include L*, a*, b*, *L*, *a*, *b*, *X*, *Y* and *Z*. Seed size traits include 7 indicators of length (s*l*), width (s*w*), length-to-width ratio(*l/w*), perimeter (s*p*), diameter (s*d*), area (s*a*) and circularity (s*c*)

The gene action model (GAM) includes additive (A), partial dominance (PD), dominance (D), over-dominance (OD)

‘R^2^’ indicates the contribution rate of the locus to the phenotype

Check marks in the last two columns indicate that the QTL was detected at a specific trial site; HN, Hainan field trial location; YY, Yuanyang field trial location

In total, 14 QTLs were detected in eight regions of five LGs for seed size traits with contribution rates of 6.43–15.12% under one environment, and LG01 and LG11 together contained more than 64% of the QTLs. Nine of the 14 QTLs individually explained the phenotypic variation of more than 10% for seed size with LOD > 3 (Table 6), while most of them presented negative additive effects of the alleles in Ganzhi 6, except for *qsc1* and *qsc2*. The pleiotropic effects in other QTLs were also identified with their close or identical locations, especially for QTLs with higher contribution rates. The collocated QTL regions were found in four regions of four LGs for the seed size traits, which contained more than one major locus. Especially, LG11 at 165.41 cM contained four QTLs with R^2^ > 10%; all loci with negative additive effects were derived from the male parent. Meanwhile, *qsp11* was distributed at 164.51 cM on LG11, with the phenotype explanation rate of 13.97%.

As to seed coat color trait, we detected 14 QTLs (Table 6). All these 14 QTLs were anchored at seven regions on three LGs; of those, eight QTLs showed high explanation value of ≥10.0%. Positive additive effects of the 14 loci were contributed by the alleles of Gaoyou 8 and explained 5.58–33.25% of the phenotype variation. On LG04, three loci were found, whereas at least two (or more) QTLs for seed coat color-related indexes were collocated at 78.19 cM, 79.58 cM, and 80.89 cM. Two QTLs, qscca*9 and *qsccZ9*, closely located on LG09. Thus, these sites might have multiple effects on the sesame seed coat color (Table 6).

Moreover, the QTLs *qtsw4* and *qtsw9*, accounting for 15.09 and 6.90% of the phenotype explanation, respectively, were detected for TSW with LOD > 3 in the HN field. The two loci contributed with negative additive effects for the alleles of the male parent. In contrast, *qtsw12* was derived from the female parent with a positive additive effect of 14.17% and explained 19.56% of the phenotypic variation under YY field conditions (Table 6).

### Candidate genes annotation

To determine the candidate genes related to the above three groups of seed traits, we screened the genes in the target intervals of stable QTLs (R^2^ ≥ 5%) (Table 7). A total of 469 genes presented within the confidence intervals. Except for two confidence intervals, one for seed coat color spaces and one for seed size traits, which had no candidate genes, the confidence intervals of all the remaining QTLs had candidate genes. In total, 439 of the 469 genes were known protein genes with functional annotation, of which, 157 were for seed coat color spaces, 116 for seed size traits, and 166 for TSW. For seed coat color traits, 108, 28, and 44 genes had been annotated using GO, KEGG, and COG, respectively. Meanwhile, 97, 32, and 48 of the 116 candidate genes for seed size traits and 123, 34, and 66 of the 166 candidate genes for TSW were annotated in GO, KEGG, and COG, respectively. In the confidence intervals of major QTLs (R^2^ ≥ 10%), 231 (52.62%) predicted genes with function annotation were screened as candidate genes for sesame seed-related traits (Tables 6 and 7).

**Table 7:** The number for the candidate genes in the confidence intervals of the stable QTLs

Genetic information				Gene number	ALL annotation	COG annotation	GO annotation	KEGG annotation
Trait	QTL	Linkage Group ID	QTL region (cM)					
Seed coat color	qsccL*4	LG04	78.19–78.68	10	10	1	8	4
	*qsccL4*							
	qsccb*4							
	qscca*4	LG04	79.58–79.99	49	48	14	34	6
	*qscca4–2*							
	*qsccL4*	LG04	80.89–81.38	2	2	0	2	2
	*qscca4–1*							
	*qsccX4*							
	*qsccY4*							
	*qsccb4*							
	qscca*9	LG09	80.47–90.56	22	22	9	15	9
	*qsccY9*	LG09	101.72–103.68	74	73	20	48	7
	*qsccZ9*	LG09	90.56–90.97	0	0	0	0	0
	*qsccZ12*	LG12	159.98–161.95	2	2	0	1	0
Seed size	*qsa1*	LG01	36.27–41.19	31	30	10	26	9
	*qsw1*							
	*ql/w1*	LG01	172.85–173.34	2	2	0	2	1
	*qsc1*	LG01	183.42–183.92	1	1	0	1	1
	*ql/w2*	LG02	16.77–17.26	0	0	0	0	0
	*qsc2*							
	*qsw5*	LG05	22.27–22.27	61	54	20	45	11
	*qsd5*							
	*qsp11*	LG11	164.51–165.41	8	8	4	6	3
	*qsa11*	LG11	165.41–166.23	11	11	6	8	3
	*qsl11*							
	*qsw11*							
	*qsd11*							
	*qsl13*	LG13	66.41–66.90	11	10	8	9	4
Thousand seed weight	*qtsw4*	LG04	57.19–57.69	18	15	5	11	1
	*qtsw9*	LG09	120.45–120.86	117	112	49	84	26
	*qtsw12*	LG12	124.45–124.92	50	39	12	28	7
Total				469	439	158	328	94

*QTLs were listed with the R^2^ ≥ 5%

As to the intervals of the seed size traits, there were 22 (5.01%) candidate genes in the flanking sequences with R^2^ ≥ 10% (Tables 6 and 7). According to the function of each gene in GO annotation information, there were 41, 20, and 56 genes involved in the cellular component category (6 branches), the molecular function category (4 branches), and the biological process category (13 branches), respectively, as some of the genes had multiple functions and could be classified into two or more function categories. Among them, most of the genes involved in the cellular component category were from the cell (12 genes), cell part (12 genes), and organelle (9 genes) categories, the majority of genes in the molecular functional category were involved in catalytic activity (10 genes) and binding (6 genes), and those in the biological process category were annotated to metabolic process (14 genes) and cellular process (12 genes) categories. In the KEGG analysis, four genes were identified in two pathways, i. e., the ribosome (ko03010) and plant hormone signal transduction (ko04075) pathways, each with two genes. In the COG analysis, a total of 22 genes were classified into seven categories in which five genes had only the general prediction function, while three functions (transcription; replication, recombination and repair; and signal transduction) had four genes each (18.18%).

For TSW, 54 (12.30%) candidate genes were functionally annotated (Tables 6 and 7). GO analysis results showed that 79 genes were detected in seven branches of the cellular component category, 46 genes in 10 branches of the molecular function category, and 101 genes in 14 branches of the biological process category. In the cellular component category, cell part, cell and organelle involved the larger number of genes with 20 genes, 19 genes and 17 genes, respectively. In the molecular function category, 21 genes and 16 genes were annotated to catalytic activity and binding, respectively. In the biological process category, 25 genes and 22 genes were annotated to metabolic process and cellular process, respectively. In the KEGG analysis, nine genes were identified in eight pathways. Two genes were found in plant hormone signal transduction (ko04075) pathway and one gene was found in other seven pathways. In the COG analysis, eight genes had only the general prediction function. Of the other 21 genes, five were related to signal transduction mechanisms, and four were related to both transcription and replication mechanism and recombination and repair mechanism. Meanwhile, three genes were related to posttranslational modification, protein turnover, and chaperones, and two genes to lipid transport and metabolism. No more than one gene was found in other functions in COG classification.

For the intervals related to seed coat color traits, 155 (35.31%) candidate genes were found (Tables 6 and 7). In GO analysis, 107 genes were grouped into 33 branches, comprising 296 genes identified in the cellular component category (10 branches), 101 genes in the molecular function category (7 branches), and 309 genes in the biological process category (16 branches); some of the genes had multiple functions and could be classified into two or more function categories. In the cellular component category, cell part and cell involved the larger number of genes with 74 genes and 72 genes, respectively. In the molecular function category, most genes were involved in catalytic activity (45 genes) and binding (42 genes), while most genes involved in the biological process category were annotated to metabolic process (69 genes) and cellular process (58 genes). In the KEGG analysis, 30 genes were identified in 21 pathways. KEGG enrichment analysis showed that the significantly enriched pathway was diterpenoid biosynthesis (ko00904) pathway, involving five genes (16.67%). A total of 54 genes were classified into 16 categories for COG classification and prediction analysis. Apart from the general prediction function, the four categories with the highest number of genes were transcription (6 genes, 11.11%), energy production and conversion (5 genes, 9.26%), replication, recombination, and repair (4 genes, 7.41%), and posttranslational modification, protein turnover, and chaperones (4 genes, 7.41%). No more than three genes were found in other functions in COG classification.

## Discussion

### Genetic analysis

Seed size, TSW, and seed coat color are important seed appearance traits. We chose two fully homozygous parents with significant differences in the referred traits for an intraspecific cross, constructed a new sesame genetic map with 2159 loci, explored the genetic basis for seed-related traits, and identified important QTLs. To improve the precision of genetic analyses for quantitative traits, we performed the segregation analysis of all traits using a large experimental group in two environments. The frequency distributions of phenotypic characters were analyzed between the two environments, revealing similar average values in different places.

As to seed size, significant positive correlations (*P* ≤ 0.01) between SL, SW, SP, SD, and SA were observed in the correlation coefficient analysis in the two field trials. Meanwhile, the values of SC significantly and negatively correlated with SL, SP, SD, SA and LWR (P ≤ 0.01). Seed size is an important factor of rice yield composition. The main indexes of seed size are SL, SW, seed thickness (ST), and LWR, with SL being the best indicator of seed size in rice [10, 35]. Therefore, future studies on seed size traits should include genetic models of SL and SC. As seed weight is a typical quantitative trait closely related to SL, SW, and ST [5, 12, 13], we analyzed the correlation between the seven important seed size traits and TSW. The results showed that five seed size traits (SL, SW, SP, SD, SA) and TSW were positively correlated in the yield obtained in 2014 at HN (P ≤ 0.01), but negatively correlated in that from 2015 at YY (*P* ≤ 0.05). The near-normal curve distribution of TSW, SL, SW, LWR, and SC suggested a polygene mode of the genetic control. This is the first study to show that seed size traits in sesame are complex quantitative traits that are controlled by multiple genes and are sensitive to environmental changes. Hence, it is imperative to examine seed phenotype-related traits repeatedly for reliable QTL mapping and conduct additional studies on genetic effects, heritability of the gene(s), and genomic function in order to clarify the molecular mechanisms controlling seed-related traits. Rice is a model crop in biological research and its seed size affects the yield and commercial quality [9–12]. Our study initiated the research on sesame seed size and would improve the yield and appearance quality of sesame seeds.

Seed coat color is an important agronomic trait for sesame, closely associated with biochemistry of protein and oil metabolism, antioxidant activity and the level of disease resistance [18–22]. Zhang et al. [23] reported that seed color is an intricate quantitative character in sesame, and it is mostly controlled by inherited genetic factors of two major genes and polygenes with additive-dominant-epistatic effects. In the genetic analysis, Wang et al. [24] published that black was dominant to white for sesame seed coat color. The F_1_ seeds of a cross from white and black sesame accessions were invariably black, so the black seed color was expressed by delayed inheritance or predetermination. The results in the research by Wang et al. [24] implied that black, white, and yellow are predominant in the sesame seed coat color indexes of L*, a*, and b*. In the F_3_ population derived from the inter-variety cross of Gaoyou 8 (white seed coat) and Ganzhi 6 (black seed coat), we found a positive and negative transgressive segregation in most seed color spaces. The seed coat color was exhibited in a typical quantitative manner, and the phenotypic variations of nine indicators related to seed coat color were significantly correlated, suggesting an important role of the genetic factor in this trait. Seed color spaces, such as L, a, b, L*, a*, b*, and so on, showed a bimodal distribution, indicating the involvement of major effect genes, which was consistent with the previous studies [23, 24]. The number of genes regulating the seed coat color trait varies in different crops, and a range of one to three genes are detected for controlling the trait [36–42]. The seed color trait is relatively stable and is not influenced by environmental factors [43–45]. In our progressive Sesame Genome Project (www.sesamum.org) [2], the research on gene clone, further genome organization and function is essential for the study of the relevancy between seed coat color and significant biochemical processes [18–22].

### Genetic linkage map

QTL mapping, with the aid of high-resolution genetic linkage maps, is always applied to locate the candidate genes associated with specific traits [26, 46]. Sesame is a self-pollinated crop, and all sesame accessions are derived from the only cultivated sesame species *S. indicum* [47–50]. Therefore, sesame has a narrower genetic base, and fewer universal molecular markers with polymorphisms can be used in sesame, such as simple sequence repeats and SNPs [23, 33, 47, 51]. Genotyping by sequencing is a high-throughput technique employed to generate enough polymorphic markers in a short time for high-density genetic map construction. Four SNP maps for sesame have previously been constructed based on reduced representation genome sequencing using the SALF-seq and restriction site-associated DNA sequencing (RAD-seq) techniques [24, 29, 31, 33].

SNPs have become the ideal marker type of choice in many evolutionary and ecological because they are the most abundant and frequent form of genetic variation in genomes [52]. In this study, we constructed a high-density genetic linkage map for sesame using the SLAF-seq approach, which has been developed recently to achieve the first rapid mass identification of SNP and InDel markers for sesame. SLAF is measured by sequencing the paired-ends of the sequence-specific restriction fragment length, while RAD sequence is measured by randomly interrupted genomic DNA after the restriction enzymes. Therefore, SLAF shows better repeatability than the RAD, and can generate large amounts of sequence information and handle whole genome density distributions. Meanwhile, this technology creates a balance between higher genotyping accuracy and relatively lower sequencing cost. In this study, we discovered more than 2000 SNPs; the rate of SNPs was 6.46% across the genome, which was higher than 5.12% reported by Zhang et al. [33]. Most SNPs belonged to the R (A/G) (32.85%) and Y (C/T) (30.43%) types, which was consistent with the observations previously published in sesame [31, 33] and other species, including even humans [53]. This new map possesses more preferable features than other published genetic maps in sesame using the similar technique, e.g., (1) larger F_2_ segregating populations of 122 individuals were applied; (2) the map was comparatively saturated with 2159 markers, including 2142 ‘SNP_only’ type; (3) the 13 LGs matched the 13 chromosomes in the sesame karyotype (2n = 26) and therefore can be used for fine genome assembly; and (4) the estimated sesame genome is 2128.51 cM, significantly longer than previously published maps of 1216 cM and 1474 cM, and with the higher average marker distance of 0.99 cM. Therefore, the map in this study was superior to the two older maps for sesame, and will contribute to the development for QTL/gene mapping, comparative genomics research, map-based cloning, and so on. Because the genetic map is constructed and integrated mainly on SNP produced by only two sesame varieties with the SNP flanking sequence of 80 bp, the utility of this map has limitations as a general tool for the research community. Due to the linkage disequilibrium (LD) decay distance (150 kb) and genome size (369 Mb by flow cytometry) [23], an ideal saturated genetic map should be based on more than 2500 SNPs, evenly distributed on each of the 13 LGs in sesame. In 2016, Zhang et al. [32] employed a genome re-sequencing strategy to construct an ultra-dense SNP genetic map for sesame in which the average marker density is approximately one bin per 0.98 cM or one SNP per 0.1 cM.

In many studies, segregation distortion has been reported to be a universal phenomenon and can be favorable for the QTL analysis if used appropriately [54–56]. Firstly, we constructed a core genetic map using Mendelian markers. Afterwards, segregation distortion markers were separately inserted into the existing map and an iterative process for the recombination fractions between markers was performed with the similar approach to that used by Wang et al. [57]. As a result, 343 markers (15.89%) with distorted segregation were mapped onto the final map with a distribution near the end of 10 out of the 13 LGs, while 79 (10.91%) [23], 205 (16.63%) [33], and 115 (9.35%) [31] markers were found on other genetic maps. Eleven SDRs were detected on nine LGs in our map, while 18 SDRs on 11 LGs [33] and four SDRs on four LGs [31] were found in other SLAF maps.

### QTL identification of seed-related traits

In this study, QTL analysis of 17 important seed traits in sesame (nine seed color spaces, seven seed size traits, and TSW) identified 31 associated regions. Nineteen QTLs contributed above 10% of the phenotypic variation with LOD > 3, of which eight QTLs were for seed coat color, nine QTLs for seed size, and two QTLs for TSW.

In the QTLs for seed coat color, *qsccY9*, *qsccZ9*, qsccb*4, and qscca*9 played major roles, explaining 33.25, 32.88, 23.10, and 20.02% of the phenotypic variation, respectively, while qscca*4, *qscca4–1*, *qscca4–2*, and *qsccb4* were regarded as polygenes due to their comparatively lower contributions. Significant correlations were detected for most of seed coat color spaces in our study, which indicated that closely linked or pleiotropic genetic factors must be the ones controlling this sort of traits. Three pleiotropic loci for seed coat color were detected on LG04 (78.19 cM, 79.58 cM, 80.89 cM) and contained 10, 49, and 2 candidate genes, respectively. Especially, qscca*4 and *qscca4–2* were collocated at 79.58 cM, explaining 19.09 and 19.79% of the phenotype variation, respectively, with LOD > 10, which was consistent with the analysis from classical genetic analysis. Other major pleiotropic seed coat color sites were located on LG09. The genes qscca*9, qsccZ9, and *qsccY9* were located closely, showing the R^2^ values more than 20%. Heritability, marker density, and sample size determine independence between two QTLs [58]. When the heritability of a QTL is 10%, marker density is 15 cM, and sample size is 300 in F_2_ or F_3_ populations, two QTLs would be adjacent with the likelihood of 80%. qscca*9, *qsccZ9*, and *qsccY9* detected in LG09 in our study are quite close to each other, with a QTL distance of about 10 cM, heritability greater than 20%, and marker density of 1.11 cM. Hence, we estimated that these QTLs may be three parts of one larger QTL in the LG09 region. We will continue to study the genetic effects and heritability of the gene(s) to further confirm this hypothesis. The high contribution rate of the locus for sesame seed coat color to phenotype was also reported by Wang et al. [24] and Zhang et al. [23]. Wang et al. [24] detected nine QTLs for seed coat color with contribution rates of 3–46%, located on four loci of LG04, LG08, and LG11. *qSCl-4.1*, a pleiotropic site for the L*, a*, and b* color space values, was identified on chromosome 4 of a 199.9-kb region, which was distributed between the bin markers SLG4_bin63 (50.4 cM) and SLG4_bin64 (50.9 cM) and contained 32 candidate genes. Zhang et al. [23] detected four QTLs of sesame seed coat color with contribution rates ranging from 9.6 to 39.9%, but the loci were mainly located with applied fragment length polymorphism markers on an independent genetic map. So we can not determine their relationship to the loci presented herein.

Finally, nine QTLs for seed size and two QTLs for TSW were detected. These are the first reported seed size-related QTLs in sesame ,and their genetic control was mostly comprised of major QTLs with R^2^ ≥ 10%. Furthermore, significant correlations among some of the seed size-related traits might be indicative of closely linked or pleiotropic genetic factors controlling these traits. This was demonstrated by co-localization of several QTLs. The significant negative correlation was found between SC and LWR, while *qsc2* with positive additive effect from Gaoyou 8 (female parent, white) alleles and *ql/w2* with negative additive effect from Ganzhi 6 (male parent, black) alleles were closely located on LG02. Meanwhile, *qsl11*, *qsd11*, *qsa11*, and *qsw11* with negative additive effect from Ganzhi 6 (male parent, black) alleles were collocated on LG11 in a 0.82-cM region with contribution rates of 12.39–15.12%, which were in line with the significant positive correlation found between SL, SD, SA, and SW. Nevertheless, QTL co-localization is not equivalent to the correlation among traits due to the effect of undetected QTLs or reasons other than pleiotropy or linkage. Many efforts have been made to accurately dissect the linked QTLs to explain these subsistent contradictions, or screen germplasm with excellent allelic variations to facilitate breeding.

Seed characters are one of the most crucial factors for sesame yield and quality. We have identified important QTLs for seed coat color, seed size, and weight in this study and have laid a preliminary foundation for marker assisted selection toward the seed-related traits in sesame. All the QTLs for seed coat color in our report showed positive additive effects deriving from the alleles of Gaoyou 8 (female parent, white), whereas most QTLs related to seed size showed negative additive effects contributed by Ganzhi 6 (male parent, black). These results will improve the breeding potential for seed-related characters and provide an efficient system for the research on series of important agricultural traits, such as yield, oil or protein content in seed, and others.

### Candidate gene function analysis

In total, there were 231 candidate genes in the confidence interval with R^2^ ≥ 10% (Tables 6 and 7). For the seed coat color trait, there were 16 function categories in COG analysis. The transcription and energy production and conversion harbored the larger number of candidate genes. In KEGG analysis, the first two pathways which harbored the largest number of genes were diterpenoid biosynthesis and oxidative phosphorylation, harboring five genes and two genes, respectively. Chang et al. [59] found that alcohol extracts of sesame peel have antioxidant components, which may be phenolic substances and terpenoids. Oxidative phosphorylation is an important energy conversion process, driving ATP synthesis. We predicted 49 and 74 candidate genes for the confidence intervals of qscca*4 and *qsccY9*, respectively, some of which may function in the regulation of seed coat color. For qscca*4, the annotation genes mainly included glutamate dehydrogenase (SIN_1016757), E3 ubiquitin protein ligase (SIN_1012044 and SIN_1012049), polyphenol oxidase (SIN_1016759), ATP binding protein (SIN_1016750), transcription elongation factor A protein (SIN_1012051), pentatricopeptide repeat-containing protein (SIN_1012034), etc. According to previous studies, SIN_1016759, and SIN_1012034 (Additional file 4: Table S4) may be related to the formation of seed coat color [17, 60, 61]. Seed color variation is involved by diverse pigments including flavonols, proanthocyanidin (carotenoid content condensed tannin), and potentially other phenolic compounds such as lignin and melanin [62]. Polyphenol oxidase (*PPO*) emerged in the oxidation step of proanthocyanidin, lignin, and melanin biosynthesis, and then resulted in a dark seed coat color [63, 64]. Many genes that encoded the enzymes in flavonoid biosynthesis have been cloned from *Arabidopsis* [65], grape [66], and soybean [67]. Wei et al. [17] reported the gene encoding *PPO* (SIN_1016759) to regulate the black color for the sesame seed coat. The pentatricopeptide repeat (PPR) family was found through a bioinformatic screening of *Arabidopsis* proteins [68], and over 450 genes encoding PPR proteins are detected in the *Arabidopsis* and rice genome [69]. Most of the PPR proteins are predicted to be targeted to either mitochondria [70, 71] or chloroplasts [72–75]. PPR proteins may also control the plant circadian rhythm [76]. The gene that encodes PPR protein may be related to the pericarp color trait of pumpkin (*Cucurbita moschata* Duch.) [77]. For site *qsccY9*, SIN_1022635 was predicted to encode an ammonium transporter, SIN_1022704 encoded a lipoxygenase (LOX), and SIN_1022679 and SIN_1022680 were identified to encode protein COBRA (Additional file 4: Table S4). These genes may contribute to seed coat color [78–80]. LOX are a family of non-heme, iron containing dioxygenases that catalyze the hydroperoxidation of polyunsaturated fatty acids, containing cis, cis-1,4-pentadiene structure, into hydroperoxides that are considered to be bitter- and grassy-flavored precursors [81–84]. In 1928, Bohn and Haas found that additions of small amounts of soybean flour to wheat flour dough decreased the normal yellow color of wheat [85]. Subsequently, it was discovered that LOXs were involved in this color loss [86]. Since then, many food scientists and soybean breeders have been interested in the bleaching activity of soybean seed LOXs on *β*-carotene or in the detection of LOX isoenzymes in soybean seeds using *β*-carotene [84, 87]. Recently, the studies on COBRA protein have been focused on some model plants. COBRA protein is closely related to the biosynthesis of cell walls in plant root, stem, and leaf tissues, and the gene’s functions have been explored. COBRA is a member of plant-specific glycosylphosphatidylinositol (GPI)-anchored protein family and plays an important role in controlling cellulose content of cell wall and orientation of cell expansion [88–91].

For the seed size and TSW, there were seven and nine function categories in COG analysis, respectively. The replication, recombination and repair, signal transduction mechanisms, and transcription harbored the largest number of candidate genes. In KEGG analysis, the plant hormone signal transduction pathway harbored two genes for both seed size and TSW, while the ribosome pathway harbored two genes for seed size. The signaling pathways of plant hormones do not act alone in plant defense responses, but there exists a cross-talk between these pathways, which is an efficient strategy to resist the invasion of different kinds of pathogens [92]. Furthermore, the ribosomes are involved in protein synthesis and regulation of some plant hormones such as auxin and gibberellin. These genes could contribute to disease resistance and plant growth and eventually to seed-related traits, particularly TSW. We checked the pleiotropic site of sesame seed size with higher explanation rates and found 11 candidate genes within the confidence intervals of *qsa11*. Annotation of these genes revealed their potential function in regulating seed size. For example, SIN_1003683 was predicted to encode a mitogen-activated protein kinase (MAPK), SIN_1003684 encoded a 50S ribosomal protein, and SIN_1003687 was identified to contain a serine/threonine-protein kinase (STK) (Additional file 4: Table S4). STK acts as an essential component of the MAPK signal transduction pathway. Depending on the cellular context, the MAPK/extracellular signal-regulated kinase cascade regulates transcription, translation, and cytoskeletal rearrangements, and then mediates diverse biological functions such as cell growth, adhesion, survival, and differentiation [93]. Ribosomal proteins are the main components of ribosomes and play an important role in the biosynthesis of proteins in cells. Ribosomes have been found to be involved in extracellular functions such as DNA repair, cell development and regulation, and cell differentiation [94]. For site *qsw5*, SIN_1013822, SIN_1013833, and SIN_1013848 may be closely linked to the seed size (Additional file 4: Table S4). In mammalian cells, a family of mitochondrial transcription termination factors (MTERFs) regulates mitochondrial gene expression. Recently, several mitochondrial regulators encoded by nuclear genome have been identified. MTERF2 is a mitochondrial matrix protein that binds to the mitochondrial DNA. Overexpression of MTERF2 can prohibit cell proliferation [95], but the mechanism has not been well defined so far. MTERF3 is the most conserved member of the MTERF family with the negative regulation on mammalian mitochondrial DNA transcription [96]. DEAD-box helicases are fundamental in DNA and RNA metabolism such as replication, repair, recombination, transcription, translation, ribosome biogenesis, and splicing, which regulate and control plant growth and development [97, 98]. Auxin response factors (ARFs) may play important roles in abscission or many auxin-mediated processes, because ARFs are transcription factors that bind to auxin response elements in promoters of early auxin response genes [99, 100].

Finally, we checked the pleiotropic site of sesame TSW and detected 117 candidate genes within the confidence intervals of *qtsw9*. SIN_1022989, SIN_1023052, and SIN_1022987 may be related to the TSW (Additional file 4: Table S4). The trihelix transcription factor family is a small family which plays an essential role in regulating plant growth and development [101] and may also be involved widely in plant responses to biological and abiotic stresses [102, 103]. At present, the trihelix protein genes have been studied in *Arabidopsis* [104], tobacco [105], and rice [106]. With the development of functional genomics, many genes that affect seed weight (size) have been successfully cloned for important crops (rice) and model plants (*Arabidopsis*) by means of mutant analysis, map-based cloning, gene expression analysis, and functional verification. These genes regulate many biochemical and genetic metabolic pathways, including carbohydrate metabolism [35], ubiquitin-proteasome [107, 108], Ser/Thr phosphatase [109], etc. Until now, Ranocha et al. [110] reported that *WALLS ARE THIN1* (*WAT1*) is a unknown tonoplast-localized auxin transporter, implying a crucial role of the vacuole in adjusting intracellular auxin homoeostasis in plants. Furthermore, exogenous auxin application can rescue the reduction in secondary cell wall thickenings in *wat1* mutants, which means that auxin may play an essential role in secondary growth for plants. Further study may be expected to verify their functions. The QTL mapping warrants further analysis to locate the genes that are nearest to the markers with highest LOD values and confirm the function of those genes in sesame seed-related traits.

## Conclusions

In summary, the present study provides the first QTL mapping of seed-related traits with an F_2_ population for sesame. We detected a total of 3129 SNPs markers using SLAF-seq sequencing and constructed a high-density genetic map in combination with InDel markers that contained the same number of linkage groups as the number of sesame chromosomes. QTL analysis was carried out on 17 important seed-related traits, and 19 major-effect QTLs individually explained more than 10% of the phenotypic variation with LOD > 3; of those, eight QTLs related to seed coat color spaces, nine QTLs related to seed size traits, and two QTLs related to TSW. Among them, 10 QTLs were detected with similar QTL regions and partially explained the correlations among these seed-related traits. *qsc2* (contributed by the female parent) and *ql/w2* (contributed by the male parent), *qscca4–1* and *qsccb4* (contributed by the female parent), and *qscca4–2* and qscca*4 (contributed by the female parent) were collocated. Four QTLs, *qsl11*, *qsd11*, *qsa11* and *qsw11*, contributed by the male parent, were located closely on LG11. A total of 469 candidate genes were identified in the confidence intervals of stable QTLs (R^2^ ≥ 5%), including 231 candidate genes for major QTLs with R^2^ value ≥10%. This report presented a solid foundation for downstream genetic analyses of seed-related traits in sesame, such as the map-based gene cloning, marker-assisted selection, and genome sequence assembly.

## Methods

### Plant material and cultivation

Two sesame varieties, Gaoyou 8 (female parent, white seeded) and Ganzhi 6 (male parent, black seeded), were used to construct a cross population. The two parents were also distinct in TSW and seed length, and Gaoyou 8 exhibited larger TSW and shorter seed length. The varieties were obtained from the sesame germplasm reservoir of the Henan Sesame Research Center, Henan Academy of Agricultural Sciences (HSRC, HAAS), China.

A total of 122 F_2_ seeds were randomly chosen from the harvested seeds of F_1_ plantlets, which were itself a cross between Gaoyou 8 and Ganzhi 6, and cultured at the Yuanyang Experimental Station of Henan Province (34°16′N, 112°42′E) in 2013. Young leaves of the 122 F_2_ individuals and the two parents were collected, frozen in liquid nitrogen, and stored at − 80 °C for DNA extraction.

Subsequently, 122 individual lines of the F_3_ population were cultured at the Sanya Experimental Station of Hainan Province (herein abbr. HN) (18°15′N, 109°30′E) and Yuanyang Experimental Station of Henan Province (herein abbr. YY) (34°16′N, 112°42′E) during 2014–2015 growing seasons for seed harvest and seed-related traits investigation. The Sanya Experimental Station is located in the southernmost part of China, in the region with a tropical maritime monsoon climate, while the Yuanyang Experimental Station is located in the Yellow River alluvial plain characterized by continental warm temperate monsoon climate. Each line was planted randomly in rows 5.0 m long and 0.4 m apart. Sixteen plantlets in each row were retained, while the others were removed at anthesis. During mature stage, seeds of five plantlets per line in the middle of each row were harvested and pooled.

### Seed-related traits investigation and data analysis

A total of 17 indicators of seed size, TSW, and seed coat color were applied to reflect the seed-related traits in sesame. Seed size and seed weight were measured using an SC-G Seed Analyser (Vision Detection Co. Ltd., Hangzhou, P.R. China). Seven seed size traits, namely seed length (SL, mm), width (SW, mm), length-to-width ratio (LWR, %), perimeter (SP, mm), diameter (SD, mm), area (SA, mm^2^), and circularity (SC) and TSW (g), were recorded. Seed coat color traits were investigated using ColorFlex EZ (HunterLab, Reston, VA, USA) ,and the values of nine color spaces (L*, a*, b*, L, a, b, X, Y, and Z) were recorded. The frequency distribution, standard error, and correlation coefficients of the above data were calculated using Excel 2007 and SPSS softwares, respectively.

### SLAF library construction and high-throughput sequencing

Before SLAF sequencing, the improved cetyltrimethylammonium bromide (CTAB) method [111] was applied to extract the genomic DNA from the 122 F_2_ individuals and the two parents. The quality of the extracted DNA was examined by electrophoresis in 1% agarose gels with a lambda DNA ladder standard and by using an ND-1000 spectrophotometer (NanoDrop Technologies, Wilmington, DE, USA).

The SLAF sequencing library was constructed based on the procedures [112] with minor modifications. Two enzymes, *Hae*III and *Hpy*166II (New England Biolabs, Ipswich, MA, USA), were used to digest the genomic DNA, and T4 DNA ligase (New England Biolabs, USA), ATP (New England Biolabs, USA), and duplex tag-labeled sequencing adapters (PAGE-purified; Life Technologies, Carlsbad, CA, USA) were added subsequently to incubate the resulting fragments at 37 °C. These diluted restriction-ligation DNA samples were mixed with dNTP, Q5® High-Fidelity DNA Polymerase, and primers (PAGE-purified; Life Technologies, USA), and were then subjected to polymerase chain reaction (PCR). The pooled products were electrophoresed on a 2% agarose gel. DNA fragments from 264 to 364 bp (including indexes and adaptors) in size were isolated and purified using a QIAquick gel extraction kit (Qiagen, Hilden, Germany). Pair-end sequencing (2 × 80 bp) was performed on an Illumina HiSeq 2500 platform (Illumina Inc., San Diego, CA, USA) according to the manufacturer’s instructions at Biomarker Technologies Corporation in Beijing (http://www.biomarker.com.cn).

### Sequence data grouping and genotyping

SNP identification and genotyping were performed as described by Sun et al. [112]. Reads of quality scores lower than 20 were filtered out. Raw reads were demultiplexed to each individual according to duplex barcode sequences. After the barcodes (0–3 bp) and the terminal positions (104–125 bp) were trimmed from reads, clean reads from the same samples were mapped onto the reference sesame genome sequences using SOAP software [113]. All sequences identified to the same position with over 95% identity were considered as one SLAF locus [114]. Alleles were defined in each SLAF using the minor allele frequency evaluation. SLAFs with two, three, and four allelic tags were identified as polymorphic and potential markers. SLAF loci with more than four alleles were discarded as repetitive SLAFs. The marker code of the polymorphic SLAFs was analyzed according to the genotypes of the F_2_ population and the segregation type (aa × bb).

A Bayesian approach was executed to further ensure genotyping quality [112]. A posteriori conditional probability was firstly calculated according to the allele coverage and the SNP number. Genotype scoring transformed from the probability was used to screen the high-quality markers for genetic mapping. The filter criteria were set as follows: SLAFs with more than three SNPs were filtered out; the average sequence depths were greater than 2-fold in each progeny and greater than 10-fold in the parents; and markers with > 30% missing data were filtered. Segregation distortion was examined using chi-square test.

### Linkage map construction

Considering the genotyping errors and deletions of NGS data, HighMap strategy was applied to order SLAFs markers, creat linkage groups (LGs) and correct genotyping errors [115]. Detailed minimum spanning tree (MST) map algorithm was also used to order SLAFs markers [116]. SMOOTH algorithm was used to correct the genotyping errors as per marker ordering [117]. The structures of all LGs were established according to the following procedures: Primary marker orders were determined by their location on the reference genome according to the relationship between ordered markers. Genotyping errors or deletions were corrected by SMOOTH algorithm. Then, MST map was applied to order the map, and SMOOTH was used to correct the new ordered genotypes with four or more cycles correction. The number of 13 LGs was randomly annotated. The map distance of each LG was estimated in centiMorgans (cM) using the Kosambi’s mapping function [118]. All markers with significant segregation distortion (*P* < 0.05) were initially excluded from the map as accessory markers. The region with more than three adjacent loci and significant segregation distortion (P < 0.05) was defined as a segregation distortion region (SDR) [119].

### QTL location analysis

rQTL package was used to detect the QTLs associated with each target trait. The composite interval mapping method was adopted with a walking speed of 1 cM [120]. The significance of each QTL interval was tested by a likelihood-ratio statistic test (likelihood of the odds [LOD] score). The threshold of the LOD score for significance (α = 0.01, 0.05, or 0.1) was determined independently using 1000 permutations. Based on the permutations, a LOD score of 2.0 was used as a minimum to declare the presence of a QTL in a particular genomic region. The QTL effect was determined according to the approach described by Stuber et al. [121].

### Identification of the candidate genes related to seed-related traits

We screened the flanking sequences of the SNP markers in candidate QTL intervals (R^2^ ≥ 10%) according to the physical sequences of the sesame reference genome (http://ocri-genomics.org/Sinbase_v2.0). We classified all the candidate genes according to the gene ontology (GO) analysis by the first 10 terms with the smallest Kolmogorov-Smirnov values, and identified the related pathways employing the Kyoto Encyclopedia of Genes and Genomes (KEGG) procedures by the first 10 pathways with the smallest *p* values [122].

## Supplementary information

**Additional file 1: Table S1.** Pairwise correlation coefficients between thousand seed weight and seed size traits for sesame under two environments. *Significant at *P* ≤ 0.05, **Significant at *P* ≤ 0.01; tsw, thousand seed weight; sl, seed length; sw, seed width; lwr, length-to-width ratio; sp., seed perimeter; sd, seed diameter; sa, seed area; sc, seed circularity.

**Additional file 2: Table S2.** Pairwise correlation coefficients of sesame seed coat color traits under two environments. *Significant at P ≤ 0.05, **Significant at *P* ≤ 0.01.

**Additional file 3: Table S3.** SLAF-seq data for each of the F_2_ individual and their parents.

**Additional file 4: Table S4.** The candidate genes for the seed-related traits.

## Data Availability

The datasets used and/or analysed during the current study are available from the corresponding author on reasonable request.

## Abbreviations

ARFs: Auxin response factors
cM: Centimorgan
COG: Clusters of orthologous groups
CTAB: Cetyltrimethylammonium bromide
GO: Gene ontology
GPI: Glycosylphosphatidylinositol
GWAS: Genome-wide association study
KEGG: Kyoto encyclopedia of genes and genomes
LG: Linkage group
LOD: Likelihood of the odds
LOX: Lipoxygenase
LWR: Length-to-width ratio
MAPK: Mitogen-activated protein kinase
MST: Minimum spanning tree
MTERFs: Mitochondrial transcription termination factors
NGS: Next-generation sequencing
PCR: Polymerase chain reaction
QTL: Quantitative trait locus
RAD-seq: Restriction site-associated DNA sequencing
SA: Seed area
SC: Seed circularity
SD: Seed diameter
SDR: Segregation distortion region
SL: Seed length
SLAF-seq: Specific length amplified fragment sequencing
SNP: Single nucleotide polymorphism
SP: Seed perimeter
ST: Seed thickness
STK: Serine/threonine-protein kinase
SW: Seed width
TSW: 1000-seed weight
