# Supplementary material for: A high-density genetic map constructed using specific length amplified fragment (SLAF) sequencing and QTL mapping of seed-related traits in sesame (Sesamum indicum L.)
Source: BMC Plant Biol. 2019 Dec 27;19:588. doi: 10.1186/s12870-019-2172-5 (PMC6935206; doi:10.1186/s12870-019-2172-5)
Supplement: Supplementary file 1 — Additional file 1: Table S1. Pairwise correlation coefficients between thousand seed weight and seed size traits for sesame under two environments. *Significant at P ≤ 0.05, **Significant at P ≤ 0.01; tsw, thousand seed weight; sl, seed length; sw, seed width; lwr, length-to-width ratio; sp., seed perimeter; sd, seed diameter; sa, seed area; sc, seed circularity. [file 12870_2019_2172_MOESM1_ESM.pdf]

Table S1 Pairwise correlation coefficients between thousand seed weight and seed size traits for sesame under two environments

Environment	Trait	tsw	sl	sw	lwr	sp	sd	sa	sc
**Hainan**	**tsw**	1							
	**sl**	0.286**	1						
	**sw**	0.473**	0.904**	1					
	**lwr**	-0.393**	0.281**	-0.154	1				
	**sp**	0.357**	0.987**	0.951**	0.147	1			
	**sd**	0.360**	0.986**	0.960**	0.123	0.996**	1		
	**sa**	0.397**	0.974**	0.975**	0.062	0.992**	0.996**	1	
	**sc**	0.331**	-0.454**	-0.040	-0.963**	-0.333**	-0.305**	-0.251**	1
**Yuanyang**	**tsw**	1							
	**sl**	-0.184*	1						
	**sw**	-0.200*	0.936**	1					
	**lwr**	-0.028	0.470**	0.130	1				
	**sp**	-0.186*	0.993**	0.964**	0.382**	1			
	**sd**	-0.205*	0.984**	0.981**	0.313**	0.993**	1		
	**sa**	-0.197*	0.983**	0.982**	0.307**	0.993**	0.999**	1	
	**sc**	0.064	-0.602**	-0.287**	-0.976**	-0.522**	-0.461**	-0.454**	1

*Significant at $P \leq 0.05$; **Significant at $P \leq 0.01$.

tsw, thousand seed weight; sl, seed length; sw, seed width; lwr, length-to-width ratio; sp, seed perimeter; sd, seed diameter; sa, seed area; sc, seed circularity.
