# Supplementary material for: A high-density genetic map constructed using specific length amplified fragment (SLAF) sequencing and QTL mapping of seed-related traits in sesame (Sesamum indicum L.)
Source: BMC Plant Biol. 2019 Dec 27;19:588. doi: 10.1186/s12870-019-2172-5 (PMC6935206; doi:10.1186/s12870-019-2172-5)
Supplement: Supplementary file 2 — Additional file 2: Table S2. Pairwise correlation coefficients of sesame seed coat color traits under two environments. *Significant at P ≤ 0.05, **Significant at P ≤ 0.01. [file 12870_2019_2172_MOESM2_ESM.pdf]

Table S2 Pairwise correlation coefficients of sesame seed coat color traits under two environments

Environment	Trait	L*	a*	b*	L	a	b	X	Y	Z
**Hainan**	**L***	1								
	**a***	0.776**	1							
	**b***	0.925**	0.901**	1						
	**L**	0.999**	0.762**	0.914**	1					
	**a**	0.825**	0.993**	0.920**	0.814**	1				
	**b**	0.960**	0.878**	0.993**	0.954**	0.909**	1			
	**X**	0.984**	0.732**	0.884**	0.991**	0.793**	0.933**	1		
	**Y**	0.982**	0.708**	0.873**	0.990**	0.771**	0.924**	0.999**	1	
	**Z**	0.953**	0.599**	0.780**	0.963**	0.667**	0.845**	0.975**	0.982**	1
**Yuanyang**	**L***	1								
	**a***	0.593**	1							
	**b***	0.918**	0.803**	1						
	**L**	0.999**	0.584**	0.911**	1					
	**a**	0.675**	0.992**	0.850**	0.668**	1				
	**b**	0.955**	0.760**	0.993**	0.951**	0.819**	1			
	**X**	0.985**	0.586**	0.896**	0.991**	0.675**	0.941**	1		
	**Y**	0.984**	0.549**	0.881**	0.991**	0.639**	0.930**	0.999**	1	
	**Z**	0.960**	0.410**	0.784**	0.968**	0.508**	0.849**	0.971**	0.980**	1

*Significant at $P \leq 0.05$; **Significant at $P \leq 0.01$.
