# Supplementary material for: A high-density genetic map constructed using specific length amplified fragment (SLAF) sequencing and QTL mapping of seed-related traits in sesame (Sesamum indicum L.)
Source: BMC Plant Biol. 2019 Dec 27;19:588. doi: 10.1186/s12870-019-2172-5 (PMC6935206; doi:10.1186/s12870-019-2172-5)
Supplement: Supplementary file 3 — Additional file 3: Table S3. SLAF-seq data for each of the F2 individual and their parents. [file 12870_2019_2172_MOESM3_ESM.pdf]

Table S1 Pairwise correlation coefficients between thousand seed weight and seed size traits for sesame under two environments

Environment	Trait	tsw	sl	sw	lwr	sp	sd	sa	sc
**Hainan**	**tsw**	1							
	**sl**	0.286**	1						
	**sw**	0.473**	0.904**	1					
	**lwr**	-0.393**	0.281**	-0.154	1				
	**sp**	0.357**	0.987**	0.951**	0.147	1			
	**sd**	0.360**	0.986**	0.960**	0.123	0.996**	1		
	**sa**	0.397**	0.974**	0.975**	0.062	0.992**	0.996**	1	
	**sc**	0.331**	-0.454**	-0.040	-0.963**	-0.333**	-0.305**	-0.251**	1
**Yuanyang**	**tsw**	1							
	**sl**	-0.184*	1						
	**sw**	-0.200*	0.936**	1					
	**lwr**	-0.028	0.470**	0.130	1				
	**sp**	-0.186*	0.993**	0.964**	0.382**	1			
	**sd**	-0.205*	0.984**	0.981**	0.313**	0.993**	1		
	**sa**	-0.197*	0.983**	0.982**	0.307**	0.993**	0.999**	1	
	**sc**	0.064	-0.602**	-0.287**	-0.976**	-0.522**	-0.461**	-0.454**	1

*Significant at $P \leq 0.05$; **Significant at $P \leq 0.01$.

tsw, thousand seed weight; sl, seed length; sw, seed width; lwr, length-to-width ratio; sp, seed perimeter; sd, seed diameter; sa, seed area; sc, seed circularity.

Table S2 Pairwise correlation coefficients of sesame seed coat color traits under two environments

Environment	Trait	L*	a*	b*	L	a	b	X	Y	Z
**Hainan**	**L***	1								
	**a***	0.776**	1							
	**b***	0.925**	0.901**	1						
	**L**	0.999**	0.762**	0.914**	1					
	**a**	0.825**	0.993**	0.920**	0.814**	1				
	**b**	0.960**	0.878**	0.993**	0.954**	0.909**	1			
	**X**	0.984**	0.732**	0.884**	0.991**	0.793**	0.933**	1		
	**Y**	0.982**	0.708**	0.873**	0.990**	0.771**	0.924**	0.999**	1	
	**Z**	0.953**	0.599**	0.780**	0.963**	0.667**	0.845**	0.975**	0.982**	1
**Yuanyang**	**L***	1								
	**a***	0.593**	1							
	**b***	0.918**	0.803**	1						
	**L**	0.999**	0.584**	0.911**	1					
	**a**	0.675**	0.992**	0.850**	0.668**	1				
	**b**	0.955**	0.760**	0.993**	0.951**	0.819**	1			
	**X**	0.985**	0.586**	0.896**	0.991**	0.675**	0.941**	1		
	**Y**	0.984**	0.549**	0.881**	0.991**	0.639**	0.930**	0.999**	1	
	**Z**	0.960**	0.410**	0.784**	0.968**	0.508**	0.849**	0.971**	0.980**	1

*Significant at P ≤0.05; **Significant at P ≤0.01.

Table S3 SLAF-seq data for each of the F_2 individual and their parents

Individuals		Q20 (%)	Q30 (%)	GC (%)	Read numbers	Average coverage	Number of SLAF markers	Read numbers for SLAFs
**Gaoyou 8 (female)**		90.69	83.95	40.36	11526675	37.60	133354	5014023
**Ganzhi 6 (male)**		89.66	82.48	40.93	10664675	35.97	123679	4448310
**F_2**	1	89.51	82.35	41.01	831785	3.77	94243	354869
	2	90.46	83.67	40.94	582966	3.08	79938	246030
	3	90.84	83.93	40.76	944539	4.55	95948	436120
	4	90.23	83.29	40.72	910610	4.49	93356	418912
	5	90.52	83.67	40.78	1096002	4.76	96168	457750
	6	89.91	82.77	41.02	804341	3.76	93265	351060
	7	90.12	83.29	40.84	723452	3.48	88707	308415
	8	90.35	83.45	40.59	946211	4.25	95144	404796
	9	88.09	80.91	41.56	381586	2.17	65255	141823
	10	89.99	83.05	40.74	1154563	4.99	99117	494338
	11	88.59	81.30	41.47	547276	2.79	80031	223437
	12	90.24	83.47	41.10	693079	3.08	83765	258404
	13	90.61	83.85	40.37	1177656	5.36	98799	529319
	14	90.31	83.46	40.64	889144	4.27	92744	395786
	15	88.89	81.43	41.21	613386	3.04	83152	252680
	16	89.40	82.28	40.60	1007807	4.59	97943	449152
	17	90.30	83.56	41.22	579676	2.86	82311	235600
	18	88.62	81.33	41.25	493450	2.69	75001	201729
	19	89.82	82.59	40.74	756499	3.76	91566	344132
	20	87.49	79.63	41.21	572630	2.76	77426	213785
	21	90.50	83.64	40.30	926109	4.46	91013	406224
	22	90.24	83.41	40.75	796147	3.73	92406	344675
	23	87.66	80.31	40.85	446502	2.35	67003	157788
	24	87.90	80.09	41.02	606200	3.08	84806	261010
	25	88.85	81.38	40.97	747342	3.54	87480	309632
	26	89.07	81.55	41.23	569973	2.93	78410	229581
	27	89.10	81.62	40.68	830189	3.92	87503	342878
	28	89.30	81.86	40.61	972951	4.74	96572	457626
	29	90.33	83.35	40.96	1020408	4.58	96902	443706
	30	88.15	80.94	41.24	620492	2.75	78810	216692
	31	89.54	82.49	41.14	834510	3.60	93621	336731
	32	90.92	84.28	40.98	939657	3.79	98355	373049
	33	90.51	83.54	40.71	719186	3.55	84447	300116
	34	87.38	79.77	40.92	860730	3.67	98341	361046
	35	89.48	82.34	40.94	1031968	4.77	100327	478393
	36	90.24	83.34	40.76	834442	3.84	91992	353084
	37	90.83	83.94	40.39	1034357	4.55	94490	430316
	38	89.99	82.89	40.54	857542	4.09	90762	371154

39	91.05	84.37	40.58	623846	3.15	83853	263975
40	88.91	81.09	40.61	999548	4.27	95935	409461
41	89.34	81.75	40.49	969038	4.43	92437	409739
42	90.42	83.40	40.71	958439	4.53	96651	437521
43	90.96	84.10	40.21	974598	4.33	93906	406542
44	89.32	82.31	40.84	516354	2.76	75704	208793
45	85.39	77.75	43.19	424726	2.11	61865	130508
46	89.46	82.45	40.51	1255700	5.37	106727	572936
47	84.37	77.13	41.75	320046	1.97	50181	98712
48	90.48	83.60	40.62	1018871	4.59	94716	434629
49	88.62	80.83	40.46	985148	4.49	95769	429937
50	90.09	82.95	40.53	1172088	4.79	97366	466741
51	90.46	83.46	40.46	1392355	5.52	105603	582541
52	91.24	84.62	39.76	1242179	5.79	97334	563084
53	91.11	84.38	40.44	1458974	6.43	106126	682816
54	89.79	82.83	40.42	1645522	6.61	106958	707422
55	90.74	83.99	40.54	1478458	6.58	103083	678363
56	88.84	81.59	40.80	1045902	4.62	96989	447899
57	89.74	82.74	40.22	1186831	5.25	97354	511389
58	89.96	83.13	39.93	1667830	7.13	106194	757413
59	89.69	82.79	40.14	1467880	6.63	101982	675705
60	90.54	83.74	39.94	1716104	7.88	101296	798502
61	89.52	82.37	40.24	1302480	5.68	103607	588287
62	91.55	84.91	40.20	1236630	5.90	96571	569545
63	90.53	83.79	40.28	1523702	6.81	103767	706955
64	90.38	83.56	40.01	1243520	5.36	94004	503973
65	91.16	84.46	39.91	2119037	9.29	102621	953013
66	90.32	83.49	40.01	1372916	6.28	102764	645802
67	89.84	82.76	40.30	1585389	6.45	106961	690235
68	90.62	83.89	40.25	1613027	6.75	106876	721450
69	90.40	83.49	40.33	1306966	5.69	104515	594606
70	91.13	84.48	40.62	988449	4.18	97560	407552
71	90.96	84.28	40.02	1402607	6.63	100335	665565
72	90.77	83.98	40.17	1001654	4.85	95748	464814
73	90.09	83.22	40.69	1349112	6.17	104581	645050
74	90.01	83.01	40.08	1568318	6.83	98219	671086
75	90.45	83.59	40.31	1686604	7.04	101884	717452
76	90.76	83.84	40.20	1362713	6.38	101280	646162
77	89.99	82.76	40.42	956251	4.17	96047	400044
78	89.42	81.70	40.27	1148639	5.08	95750	486589
79	90.67	83.68	39.93	1281933	6.04	97056	586592
80	90.25	83.11	39.98	1584361	6.97	100674	701707
81	89.93	82.85	40.33	1566610	6.87	106632	732320
82	90.43	83.44	40.30	1696533	7.28	103094	750583

83	90.63	83.78	40.02	1198463	5.37	94737	508378
84	90.27	83.20	40.38	992497	4.53	91043	412238
85	89.53	82.40	40.28	1522623	6.32	104018	657074
86	89.56	82.38	40.24	1125607	4.91	90248	442862
87	89.52	82.38	40.86	1296257	5.16	106465	549696
88	89.86	82.76	40.24	1435622	5.82	91407	531923
89	90.30	83.35	40.42	1235114	5.48	95590	523576
90	90.64	83.78	40.51	1459885	5.89	103272	608513
91	90.53	83.70	40.64	1297479	5.24	104945	549963
92	90.89	84.12	40.00	1271354	5.92	92812	549294
93	90.39	83.42	40.30	1400892	6.33	100741	637845
94	90.57	83.70	40.15	1540189	6.85	101774	697218
95	89.15	81.72	40.19	1638846	7.04	103861	731663
96	89.74	82.53	40.09	1248681	5.68	99614	565980
97	91.29	84.46	40.20	1203427	5.97	99345	593250
98	90.22	83.19	40.17	1418995	6.29	101615	639294
99	90.38	83.33	40.16	1095829	5.04	96874	487824
100	90.53	83.57	40.68	1515998	6.44	110277	710325
101	90.47	83.49	40.21	1569362	6.69	100581	672734
102	90.48	83.55	40.44	1612570	6.93	106477	737614
103	90.87	84.11	40.05	1509447	6.77	99066	670450
104	90.90	84.02	40.32	1587542	6.45	103553	667918
105	90.73	83.74	40.12	1182958	5.35	99487	532524
106	90.24	83.15	40.29	1386637	5.67	97727	553970
107	90.33	83.10	40.16	1361840	6.07	100275	608641
108	91.37	84.54	40.19	1886076	7.92	103145	817077
109	91.07	84.41	40.50	1435393	5.89	102405	602940
110	90.61	83.87	40.39	1858483	7.61	104543	795331
111	90.30	83.41	40.27	1571502	6.71	100818	676938
112	89.90	82.69	41.06	1262074	5.30	104023	551589
113	91.44	84.63	40.06	1176685	5.62	94327	530424
114	90.20	83.18	40.22	1474670	6.21	100587	624872
115	90.37	83.40	40.16	1379990	6.02	98057	590267
116	90.40	83.36	40.50	1861126	7.56	108320	818501
117	89.58	82.21	40.69	1667411	6.69	113301	758010
118	90.25	83.27	40.57	1698482	6.65	106212	706178
119	89.89	82.55	40.78	1417296	5.87	98659	578854
120	91.71	85.00	40.41	1021925	4.99	91165	454620
121	91.05	84.28	40.62	1333219	5.58	109600	611442
122	90.77	84.03	40.51	1290453	5.74	101605	583091
