# Supplementary material for: A high-density genetic map constructed using specific length amplified fragment (SLAF) sequencing and QTL mapping of seed-related traits in sesame (Sesamum indicum L.)
Source: BMC Plant Biol. 2019 Dec 27;19:588. doi: 10.1186/s12870-019-2172-5 (PMC6935206; doi:10.1186/s12870-019-2172-5)
Supplement: Supplementary file 4 — Additional file 4: Table S4. The candidate genes for the seed-related traits. [file 12870_2019_2172_MOESM4_ESM.pdf]

Table S4 The candidate genes for the seed-related traits

Trait	QTL	Linkage Group ID	Gene ID	Swissprot annotation	Nr annotation
**Seed coat color**	*qscca*4*	LG04	SIN_1012044	E3 ubiquitin protein ligase RIN2 GN=F24A6.70 OS=Arabidopsis thaliana (Mouse-ear cress) PE=1 SV=1	PREDICTED: RING finger and transmembrane domain-containing protein 2-like [Solanum lycopersicum]
			SIN_1012049	Putative SWI/SNF-related matrix-associated actin-dependent regulator of chromatin subfamily A member 3-like 2 GN=At5g22750 OS=Arabidopsis thaliana (Mouse-ear cress) PE=2 SV=1	PREDICTED: E3 ubiquitin-protein ligase SHPRH-like [Vitis vinifera]
			SIN_1016759	Aureusidin synthase GN=AS1 OS=Antirrhinum majus (Garden snapdragon) PE=1 SV=1	polyphenol oxidase [Populus trichocarpa]
			SIN_1012034	Pentatricopeptide repeat-containing protein At1g02060, chloroplastic (Precursor) GN=At1g02060 OS=Arabidopsis thaliana (Mouse-ear cress) PE=2 SV=2	PREDICTED: pentatricopeptide repeat-containing protein At1g02060, chloroplastic-like [Solanum lycopersicum]
	qsccY9	LG09	SIN_1022635	Ammonium transporter 1 member 3 GN=AMT1-3 OS=Solanum lycopersicum (Tomato) PE=2 SV=1	PREDICTED: ammonium transporter 1 member 3 [Vitis vinifera]
			SIN_1022704	Probable linoleate 9S-lipoxygenase 5 GN=LOX1.5 OS=Solanum tuberosum (Potato) PE=2 SV=1	lipoxygenase [Olea europaea]
			SIN_1022679	Protein COBRA (Precursor) GN=COB OS=Arabidopsis thaliana (Mouse-ear cress) PE=2 SV=1	PREDICTED: protein COBRA [Vitis vinifera]
			SIN_1022680	COBRA-like protein 4 (Precursor) GN=COBL4 OS=Arabidopsis thaliana (Mouse-ear cress) PE=2 SV=2	Protein COBRA precursor, putative [Ricinus communis]
**Seed size**	*qsa11*	LG11	SIN_1003683	Mitogen-activated protein kinase YODA GN=YDA OS=Arabidopsis thaliana (Mouse-ear cress) PE=1 SV=1	protein kinase [Nicotiana benthamiana]
			SIN_1003684		PREDICTED: 50S ribosomal protein L25-like [Solanum lycopersicum]
			SIN_1003687	Serine/threonine-protein kinase SAPK10 GN=OSJNBb0007E22.11 OS=Oryza sativa subsp. japonica (Rice) PE=2 SV=1	PREDICTED: serine/threonine-protein kinase SRK2I-like [Solanum lycopersicum]
	qsw5	LG05	SIN_1013822		Mitochondrial transcription termination factor family protein [Theobroma cacao]
			SIN_1013833	DEAD-box ATP-dependent RNA helicase 5 GN=B1114D08.16-1 OS=Oryza sativa subsp. japonica (Rice) PE=2 SV=1	PREDICTED: DEAD-box ATP-dependent RNA helicase 5 [Vitis vinifera]
			SIN_1013848	Auxin response factor 9 GN=ARF9 OS=Arabidopsis thaliana (Mouse-ear cress) PE=1 SV=1	Auxin response factor 9 isoform 2 [Theobroma cacao]
**Thousand seed weight**	*qtsw9*	LG09	SIN_1022989	Trihelix transcription factor GT-3a GN=T10O8.90 OS=Arabidopsis	Sequence-specific DNA binding transcription

		thaliana (Mouse-ear cress) PE=1 SV=1	factors [Theobroma cacao]
	SIN_1023052	Serine/threonine-protein phosphatase PP2A-3 catalytic subunit GN=F14P22.90 OS=Arabidopsis thaliana (Mouse-ear cress) PE=2 SV=1	serine/threonine-protein phosphatase [Genlisea aurea]
	SIN_1022987	WAT1-related protein At1g25270 GN=At1g25270 OS=Arabidopsis thaliana (Mouse-ear cress) PE=2 SV=1	Mtn21-like protein [Theobroma cacao]
